# Thoughts on the evolution of Core Environmental Responses in yeasts

**DOI:** 10.1016/j.funbio.2020.01.003

**Published:** 2020-05

**Authors:** Alistair J.P. Brown, Daniel E. Larcombe, Arnab Pradhan

**Affiliations:** MRC Centre for Medical Mycology, University of Exeter, Department of Biosciences, Geoffrey Pope Building, Stocker Road, Exeter, EX4 4QD, UK

**Keywords:** Core stress responses, Evolution of anticipatory responses, Fungal memory, Fungal pathogens, Immune evasion, Stress responses

## Abstract

The model yeasts, *Saccharomyces cerevisiae* and *Schizosaccharomyces pombe*, display Core Environmental Responses (CERs) that include the induction of a core set of stress genes in response to diverse environmental stresses. CERs underlie the phenomenon of stress cross-protection, whereby exposure to one type of stress can provide protection against subsequent exposure to a second type of stress. CERs have probably arisen through the accumulation, over evolutionary time, of protective anticipatory responses (“adaptive prediction”). CERs have been observed in other evolutionarily divergent fungi but, interestingly, not in the pathogenic yeast, *Candida albicans*. We argue that this is because we have not looked in the right place. In response to specific host inputs, *C. albicans* does activate anticipatory responses that protect it against impending attack from the immune system. Therefore, we suggest that *C. albicans* has evolved a CER that reflects the environmental challenges it faces in host niches.

## Highlights

• We review Core Environmental Responses (CERs) in domesticated and pathogenic yeasts.
• CERs probably evolved through the accumulation of protective anticipatory responses.
• Evolutionarily diverse yeasts display CERs, but the pathogen, *Candida albicans*, does not.
• *C. albicans* has evolved an alternative CER that protects against immune clearance.
• This has implications for the investigation of CERs in other fungi.

## Graphical abstract

Domesticated yeasts such as *S. cerevisiae* display Core Environmental Responses that have evolved to provide protective anticipatory responses that confer cross-protection against diverse environmental stresses. The pathogenic yeast, *C*. *albicans*, does not appear to display stress cross-protection, but does display anticipatory responses that protect against immune clearance. This suggests that some yeasts have evolved Core Environmental Responses that reflect the environmental challenges posed by their respective niches, and that the nature of the niche should be considered when examining Core Environmental Responses.

**Figure:**
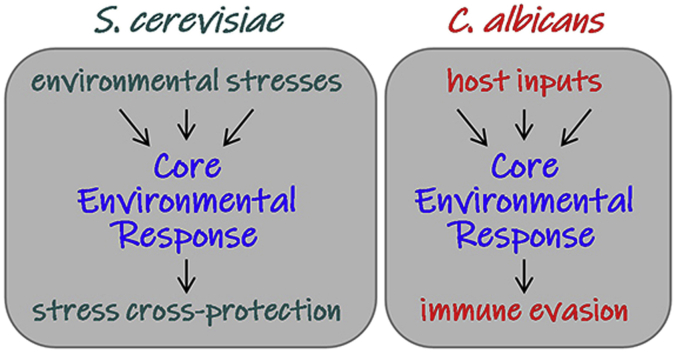

## 1 Introduction

To survive in dynamically changing niches, yeasts must be able to detect environmental challenges and activate the appropriate cellular responses. Environmental changes that perturb cellular homeostasis represent a stress to the yeast cell and, therefore, the imposition of stress is likely to be a common occurrence. Indeed, it has been suggested that microbes are unlikely ever to be completely stress-free (Hallsworth, 2018).

Stresses such as changes in ambient temperature, pH, osmolarity and redox status are likely to have influenced the evolution of ancient life forms in diverse ecosystems. Consequently, fundamental aspects of the cellular responses to these stresses are conserved across all kingdoms of life (Kultz, 2003). For example, in bacteria, archaea and eukaryotes, heat shock induces the synthesis of evolutionarily conserved protein chaperones that protect against the perturbation of cellular proteostasis (Karlin and Brocchieri, 1998, Lindquist, 1986). Furthermore, the key regulators that control this heat shock response are conserved across the fungal kingdom and eukaryotes in general, and this evolutionary conservation of key regulatory modules holds true for other stress responses, such as for osmotic and oxidative stress (Brown et al., 2017).

The presence of an Environmental Stress Response was proposed in *Saccharomyces cerevisiae* in the 1990’s (Martinez-Pastor et al., 1996). The global definition of core responses to environmental change followed the development of technologies for genome-wide transcriptional profiling in the evolutionarily divergent model yeasts, *S. cerevisiae* and *Schizosaccharomyces pombe* (Causton et al., 2001, Chen et al., 2003, Gasch et al., 2000). The Core Stress Response, or Core Environmental Response (CER), represents a set of genes that is commonly induced in response to diverse types of environmental input, plus a second set of genes that is commonly repressed in response to these inputs. CERs have since been discovered in other fungi, plants and animals (Dodd et al., 2018, Emri et al., 2015, Hahn et al., 2013, Roetzer et al., 2008, Singh et al., 2010). As discussed below, these CERs provide additional levels of cellular protection, over and above the protection provided by stress-specific signalling pathways. Therefore, the finding that the fungal pathogen, *Candida albicans*, displays a dramatically reduced CER (Brown et al., 2014a, Enjalbert et al., 2003, Enjalbert et al., 2006, Nicholls et al., 2004) was particularly interesting. This article considers why CERs might have evolved in fungi and, importantly, how these CERs might have evolved. On this basis, we suggest that *C. albicans* has probably evolved a CER after all, but that this CER has remained obscure because we have not looked in the right place!

## 2 Perspectives on Core Environmental Responses

### 2.1 Key stress signalling modules and responses are evolutionarily conserved

CERs probably evolved in fungi because they provide fitness advantages over and above stress-specific responses. These stress-specific responses are likely to have evolved earlier than CERs because, as mentioned above, they drive adaptation to fundamental environmental challenges that were, no doubt, experienced by ancient ancestral species. There exists a wide variety of stress-specific responses, which protect against xenobiotic, pH extremes, weak acids, UV and other forms of radiation, to name a few. For the purposes of this discussion, heat shock, oxidative and osmotic stress is discussed briefly here because these are often examined as part of core stress responses.

In response to heat shock, fungi induce the expression of heat shock proteins, which include chaperones that promote protein (re)folding (Karlin and Brocchieri, 1998, Lindquist, 1986). This induction is mediated by an essential, evolutionarily conserved, auto-regulatory circuit in which Hsp90 controls the activity of the heat shock transcription factor Hsf1 (Leach et al., 2012a, Nicholls et al., 2009, Taipale et al., 2010, Voellmy, 2004). In addition to promoting the adaptation and recovery of cells from an initial heat shock, this response provides transient protection against a subsequent, more severe, heat shock (Piper, 1993).

Similarly, prior exposure to oxidative stress provides fungal cells with protection against a subsequent, more severe oxidative stress (Collinson and Dawes, 1992, Davies et al., 1995). This protection is mediated by cellular adaptation to oxidative stress, which is dependent upon AP-1-like transcription factors that are conserved from yeasts to mammals (Toone et al., 2001). For example, in *S. cerevisiae, Sz. pombe* and *C. albicans*, the transcriptional induction of oxidative stress responsive genes is driven largely by the transcription factors Yap1, Pap1 and Cap1, respectively (Alarco and Raymond, 1999, Stephen et al., 1995, Toone et al., 1998, Znaidi et al., 2009). Their target genes encode functions involved in the detoxification of the oxidative stress as well as proteins that repair the damage caused by the oxidative stress (Brown et al., 2017, Cohen et al., 2002, Znaidi et al., 2009).

Hyper-osmotic stresses also trigger molecular responses in fungi that lead to cellular adaptation to this stress and transient protection against a subsequent hyper-osmotic stress (Hohmann, 2002, Klipp et al., 2005). This adaptation, which includes the accumulation of osmolytes that promote the restoration of turgor pressure and growth (Albertyn et al., 1994, Kayingo and Wong, 2005, San Jose et al., 1996), is dependent on a highly conserved mitogen activated protein kinase (MAPK) signalling module that includes the Hog1 stress activated protein kinase (Sty1 in *Sz. pombe*) (Brewster et al., 1993, Enjalbert et al., 2006, Millar et al., 1995, San Jose et al., 1996).

Stress gene expression is thought to be costly in energetic terms, in part because stress gene expression is noisy relative to housekeeping genes, for example (Lopez-Maury et al., 2008). The induction of energy generating metabolic functions in response to stress is consistent with the view that mounting stress responses is energetically demanding (Causton et al., 2001, Chen et al., 2003, Enjalbert et al., 2006, Gasch et al., 2000, Roetzer et al., 2008). Nevertheless, the cost-benefits of mounting of stress responses seem to be favourable as these types of response have been retained across all kingdoms of life. In addition to promoting stress adaptation and cellular recovery, these responses provide transient protection against a subsequent, acute dose of the same type of stress. The length of this protection depends on the rate of loss of the “molecular memory” (i.e. the protective enzymes or molecules) following the initial adaptation (Klipp et al., 2005, You et al., 2012).

The maintenance of cellular homeostasis under “normal” conditions (i.e. in the absence of stress) provides another strong selective pressure for the evolutionary retention of stress responses. Stress responses are frequently studied following the imposition of acute doses of stress. However, in reality, the heat shock response is activated and maintains proteostasis during mild temperature fluctuations (Leach et al., 2012b), the osmotic stress response is activated during subtle changes in water balance (Muzzey et al., 2009), and no doubt oxidative stress response functions promote cellular redox homeostasis in the absence of large doses of exogenous reactive oxygen species (ROS). Clearly, significant evolutionary pressures have promoted the retention of stress-specific responses.

### 2.2 Fungal Core Environmental Responses differ

Core responses to environmental change were defined comprehensively in the domesticated yeast, *S. cerevisiae,* by transcript profiling following exposure to a wide variety of stresses, including thermal, oxidative, osmotic, pH and nutrient stresses (Causton et al., 2001, Gasch et al., 2000). Hierarchical clustering of genes based on their expression patterns under these conditions revealed a large set of genes that was down-regulated under stress conditions. Many of these genes encode growth-related functions (transcription, RNA processing, translation, cell cycle), consistent with the observation that growth is temporarily slowed during cellular adaptation to stress (Escote et al., 2004). Interestingly, a core set of up-regulated genes was also observed under many of the stress conditions examined (Causton et al., 2001, Gasch et al., 2000). These included heat-shock and oxidative stress genes, as well as genes involved in central carbohydrate metabolism and energy generation. Together, these up- and down-regulated genes represent the CER in *S. cerevisiae*. The partially functionally redundant zinc-finger transcription factors, Msn2 and Msn4, are essential for the activation of up-regulated CER genes (Causton et al., 2001, Gasch et al., 2000, Martinez-Pastor et al., 1996).

The pathogenic yeast, *Candida glabrata,* also displays a CER (Roetzer et al., 2008). Like *S. cerevisiae*, the core set of up-regulated genes in *C. glabrata* includes heat shock, oxidative and osmotic stress genes, and their induction is dependent on Msn2. Interestingly, constitutive activation of *MSN2* is deleterious to *C. glabrata,* consistent with the idea that CER activation is energetically demanding (Roetzer et al., 2008).

Despite having diverged from *S. cerevisiae* and *C. glabrata* around 300 million y ago (Dujon et al., 2004), *Sz. pombe* also displays a CER (Chen et al., 2003). Once again, heat shock, antioxidant and energy generating functions were identified in the core set of up-regulated genes. However, the activation of these CER genes was dependent on Sty1 (the *Sz. pombe* orthologue of the Hog1 MAPK) and the transcription factor, Atf1 (Chen et al., 2003), rather than Msn2/4 orthologues (Causton et al., 2001, Gasch et al., 2000, Roetzer et al., 2008). Therefore, there appears to have been regulatory rewiring of the CER in *Sz. pombe* relative to those in *S. cerevisiae* and *C. glabrata* (Gasch, 2007).

The surprise came when the CER was examined in *C. albicans*. Like *C. glabrata*, *C. albicans* is a major fungal pathogen of humans. Both species are frequent causes of life-threatening systemic infections in immunocompromised patients (Morgan, 2005, Pfaller et al., 2014). However, unlike *C. glabrata* (Roetzer et al., 2008), the CER in *C. albicans* was found to be minimal, if not non-existent (Enjalbert et al., 2003, Enjalbert et al., 2006). There was minimal overlap between heat shock, oxidative stress or osmotic stress genes (Enjalbert et al., 2003), and the small set of putative CER genes (24 genes) was not significantly enriched for genes involved in oxidative or osmotic stress or energy generation (Enjalbert et al., 2006). Furthermore, the orthologues of Msn2/4, the key transcriptional inducers of the CER in *S. cerevisiae* and *C. glabrata,* have been functionally reassigned in *C. albicans* (Nicholls et al., 2004, Ramsdale et al., 2008). Therefore, the apparent lack of a broad CER in *C. albicans* is not a trivial observation based on the stress doses used in the transcript profiling experiments, for example.

### 2.3 Core Environmental Responses confer stress cross-protection

Given that CERs are likely to be even more energetically demanding than stress-specific responses (Lopez-Maury et al., 2008), the evolutionary retention of CERs in diverse yeasts suggests that these responses must confer significant fitness benefits over stress-specific responses.

The phenomenon of stress cross-protection provides one such fitness benefit. This is where exposure to one type of stress confers protection against subsequent exposure to a different type of stress. For example, exposing *S. cerevisiae* to a mild heat shock confers protection against a subsequent oxidative, osmotic or freeze-thaw stress (Lewis et al., 1995, Park et al., 1997, Wieser et al., 1991). Stress cross-protection has been shown to be dependent on new protein synthesis and upon the CER regulators, Msn2 and Msn4 (Berry and Gasch, 2008). For example, exposure to salt protects *S. cerevisiae* cells against subsequent exposure to an oxidative stress, as well as to subsequent salt exposure. Also, exposure to heat shock protects yeast cells against subsequent exposure to an oxidative stress, as well as to subsequent heat shock (Berry and Gasch, 2008). Expressing stress functions in the absence of stress does incur a fitness cost (Markiewicz-Potoczny and Lydall, 2016, Pradhan et al., 2017). Nevertheless, by conferring stress cross-protection, the CER appears to have provided a significant fitness benefit during the evolution of a number of yeasts (Berry and Gasch, 2008, Lopez-Maury et al., 2008).

### 2.4 Core Environmental Responses probably arose through the development of protective anticipatory responses

The CER might be costly in energetic terms, but it appears to confer significant fitness benefits (above). Yet there has been dramatic evolutionary rewiring of the CER in *C. albicans* relative to other ascomycete yeasts, and this does not simply relate to its pathogenic lifestyle, as both *C. albicans* and *C. glabrata* are major pathogens of humans (above). Therefore, why might the CER have been rewired in *C. albicans*? The answer to this question probably lies in an understanding of how CERs arose.

The existence of common underlying mechanisms might have contributed to the development of CERs. For example, exposure to heat shock or antifungal drugs leads an increase in intracellular ROS production levels (Abrashev et al., 2008, Davidson and Schiestl, 2001). In principle, this might explain why heat shock also activates an oxidative stress response (Causton et al., 2001, Chen et al., 2003, Gasch et al., 2000, Roetzer et al., 2008). However, this is not the case in *C. albicans* (Enjalbert et al., 2003, Enjalbert et al., 2006). Hence, the existence of common underlying mechanisms is not sufficient to explain how CERs arose.

**Fig. 1:**
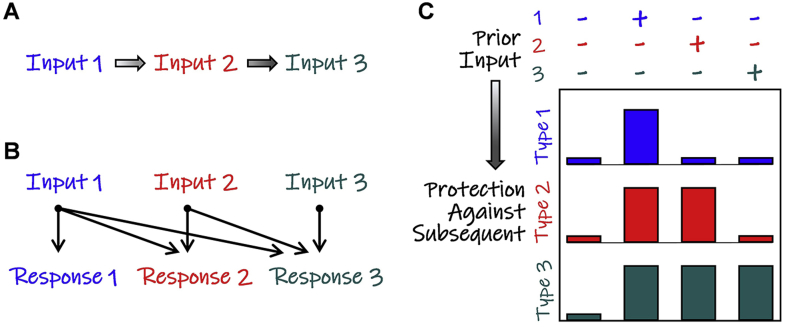
Core Environmental Responses are anticipatory responses that confer stress cross-protection. (A) Some microbes inhabit reasonably predictable environments where one type of environmental input is often followed by a second, and possibly a third. (B) Such microbes may gain a fitness advantage by activating anticipatory adaptive responses against impending inputs when the first input is detected. This phenomenon has been termed “adaptive prediction” (Mitchell et al., 2009). These authors point out that symmetric anticipatory responses can occur, in addition to the asymmetric responses shown here. (C) As a result of anticipatory adaptive responses, exposure to one environmental input can enhance protection against a subsequent environmental input of a different type – a phenomenon called “stress cross-protection”. The accumulation of anticipatory adaptive responses probably underlies the development of Core Environmental Responses (CERs) in some yeasts (see text).

The broad coverage of functions activated by the CERs in *S. cerevisiae, Sz. pombe* and *C. glabrata* (Causton et al., 2001, Chen et al., 2003, Gasch et al., 2000, Roetzer et al., 2008) is unlikely to have arisen in a single evolutionary event. This broad coverage is more likely to have developed over time via an accumulation of protective responses. Mitchell and co-workers have suggested that, during its domestication, *S. cerevisiae* has evolved in a reasonably predictable environment that imposes a reasonably predictable series of inputs (Mitchell et al., 2009). They argue that, during fermentation, the rise in temperature is followed by a switch from fermentative to respiratory metabolism, which is accompanied by changes that include elevated intracellular ROS levels. They suggest that, as a result, *S. cerevisiae* has gained a fitness advantage by developing anticipatory responses that include protection against the impending oxidative stress that often follows an increase in ambient temperature (Mitchell et al., 2009). Their ineluctable hypothesis is that microbes that inhabit reasonably predictable environments might gain a fitness advantage through “adaptive prediction” – the development of protective anticipatory responses (Fig. 1). It has been argued that such anticipatory responses represent a primitive form of microbial memory (Brown et al., 2019, Casadesus and D’Ari, 2002, Hellingwerf, 2005, Wolf et al., 2008).

**Fig. 2:**
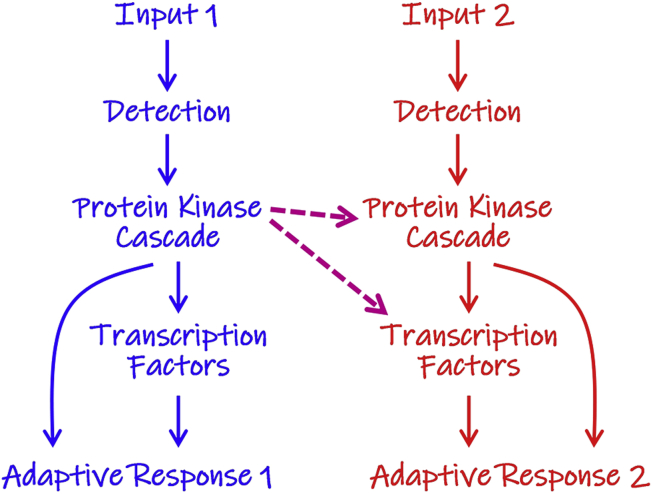
Potential mechanisms underlying the development of protective anticipatory responses. In principle, the development of a new anticipatory response, whereby exposure to one environmental input (blue) can provide cross-protection against subsequent exposure to a second type of input (red), could arise through a number of mechanisms. Arguably, the most straightforward might involve the generation of a new regulatory target site for a protein kinase from the blue pathway on a protein kinase or transcription factor on the red pathway. This new link (either of the purple arrows) could bring downstream signalling components on the red pathway under the control of upstream regulators on the blue pathway (see text). (For interpretation of the references to colour in this figure legend, the reader is referred to the Web version of this article.)

How quickly can a microbe become entrained to a repetitive environment? Microevolution experiments, involving the exposure of *Saccharomyces cerevisiae* cells to repetitive environmental inputs, have revealed that this yeast can rapidly develop anticipatory responses, within 50–150 generations (Dhar et al., 2013, Lopez Garcia de Lomana et al., 2017). In principle, this could be straightforward from a mechanistic point of view (Fig. 2). For example, a signalling pathway could develop control over an alternative stress regulon simply through the emergence of a new protein kinase target site on a regulatory protein (Bleuven and Landry, 2016, Holt et al., 2009).

Therefore, fungal CERs have probably developed through the accumulation of protective anticipatory responses. To some degree, these CERs may reflect ancient, common molecular mechanisms that underlie responses to certain types of stress. However, given the speed with which regulatory links can evolve, at least *in vitro* in response to tightly defined environmental transitions, it seems likely that CERs have undergone evolutionary tuning in response to the patterns of stress imposition by their niche. Therefore, the nature of the niche might account for the rewiring of the CER in *C. albicans*.

### 2.5 A Core Environmental Response in a yeast that is obligately associated with warm-blooded animals

*C. albicans* is a commensal of humans. However, this fungus often causes mucosal infections in otherwise healthy individuals, and life-threatening systemic infections in immunocompromised patients (Brown et al., 2012, Denning et al., 2018, Neville et al., 2015). *C. albicans* has been isolated from animals as well as humans (Odds et al., 1988). It has also been recovered from environmental samples including plants, soil, lakes, sewage and hospital laundry (Barnett et al., 1983, Bensasson et al., 2019, Gentles and La Touche, 1969), but these environmental isolates have been restricted mainly to sites that may have been contaminated by humans or animals (Odds et al., 1988). Consequently, *C. albicans* is considered to be obligately associated with warm-blooded animals (Odds et al., 1988). Herein might lie the basis for the divergence of the CER in *C. albicans* compared with *S. cerevisiae* and *Sz. pombe*. *C. glabrata* might also lack an environmental reservoir (Silva et al., 2012), and yet this pathogen has retained a CER (Roetzer et al., 2008). However, the shorter evolutionary distance between *C. glabrata* and *S. cerevisiae,* compared with *C. albicans* (Shen et al., 2016), means there has been less time for the CERs of *C. glabrata* and *S. cerevisiae* CER to have diverged. In other words, in principle, the CER of *C. albicans* has had more time to be tuned to the evolutionary pressures of host niches. On this basis, should *C. albicans* still display a CER, we have probably been looking for this CER in the wrong place! Should it exist, this CER is more likely to reflect the evolutionary pressures of host niches.

What types of pressures does *C. albicans* face in host niches? These pressures include our immune system, and innate immunity in particular [which normally clears *C. albicans* efficiently from the bloodstream and tissues (Dambuza and Brown, 2015, Netea et al., 2015)], host-imposed nutritional immunity [which attempts to deprive the fungus of essential micronutrients such as iron and zinc (Crawford and Wilson, 2015, Potrykus et al., 2013)], contrasting nutrient availabilities in different host niches [comparing the colon, vagina and bloodstream, for example (Barelle et al., 2006, Brown et al., 2014b, Childers et al., 2016, Ramirez and Lorenz, 2007)], and hypoxic microenvironments [particularly in the gastrointestinal tract and fungal lesions (Ernst and Tielker, 2009, Grahl et al., 2012, Lopes et al., 2018)]. Interestingly, certain specific carbon sources, iron deprivation and hypoxia all trigger protective responses in *C. albicans* that promote immune evasion (Ballou et al., 2016, Pradhan et al., 2018, Pradhan et al., 2019).

Innate immune cells recognise invading microbes as “foreign” via pathogen associated molecular patterns (PAMPs). *C. albicans* displays β-glucan, mannan and chitin at its cell surface, and all three are recognised as PAMPs by innate immune cells (Erwig and Gow, 2016, Netea et al., 2006, Netea et al., 2008). β-glucan, in particular, is highly inflammatory and its recognition by the pattern recognition receptor, Dectin-1, is important for antifungal immunity in mice and humans (Brown and Gordon, 2001, Ferwerda et al., 2009, Marakalala et al., 2013, Sem et al., 2016, Taylor et al., 2007, Werner et al., 2009). PAMP recognition stimulates phagocytosis of *C. albicans* cells by innate immune cells, which attempt to kill the fungus with a combination of acute stresses that include reactive oxygen, nitrogen and other chemical species, cationic stresses and nutrient starvation (Brown, 2011). The combination of stresses appears particularly effective in killing *Candida* cells (Kaloriti et al., 2014). Therefore, there must be a strong selective pressure on *C. albicans* cells to avoid recognition by innate immune cells.

**Fig. 3:**
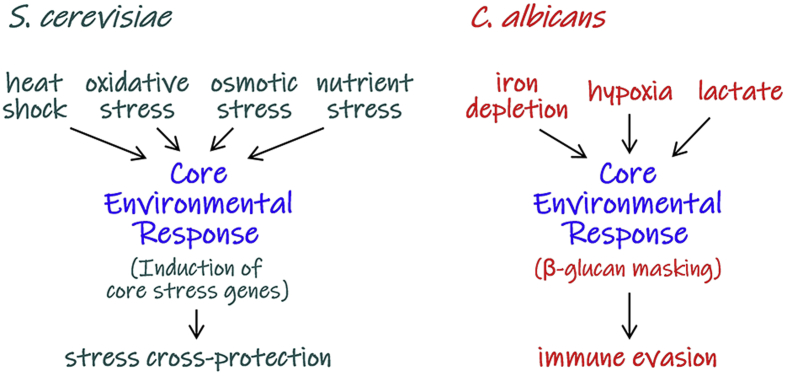
Core Environmental Responses in a domesticated and pathogenic yeast. The Core Environmental Responses (CERs) in *S. cerevisiae* can be activated by a wide range of environmental inputs that include stresses and nutrient depletion. This CER leads to the activation of core stress genes and, thereby, to protection against a variety of impending environmental challenges. By analogy, the pathogenic yeast, *C. albicans*, triggers β-glucan masking in response to a variety of specific inputs imposed by the host. This β-glucan masking attenuates recognition of *C. albicans* by innate immune cells, thereby providing some protection against immune clearance. Therefore, these domesticated and pathogenic yeasts appear to have evolved CERs that reflect the environmental challenges imposed by their respective niches (see text).

We have found that *C. albicans* evades immune recognition my reducing β-glucan exposure at its cell surface, and that it does so in response to environmental inputs that are signatures of impending immune attack. Exposure to lactate (Ballou et al., 2016), hypoxia (Pradhan et al., 2018) or iron depletion (Pradhan et al., 2019) triggers β-glucan masking at the *C. albicans* cell surface, and this correlates with a decrease in phagocytosis and attenuated immune responses. For lactate exposure (Ene et al., 2012) and hypoxia (Lopes et al., 2018), the attenuated immune response correlates with an increase in the virulence of the fungus. Therefore, *C. albicans* displays anticipatory responses that provide protection against our immune defences (Brown et al., 2019). Might this common induction of a protective response by diverse environmental inputs be the *C. albicans* equivalent of a Core Environmental Response (Fig. 3)?

## 3 Conclusion

In conclusion, Core Environmental Responses (CERs) have generally been defined on the basis of responses to “standard” experimental inputs that were developed by the model yeast research communities (e.g. Causton et al., 2001, Chen et al., 2003, Gasch et al., 2000). We suggest that, as our exploration extends into fungal pathogens of humans, animals and plants, and into saprobic fungal species, we should consider CERs in broader terms. For example, we argue that the CER of *C. albicans* includes immune evasion. For other fungal pathogens, parasites or saprobes, what types of anticipatory response might, in principle, confer fitness benefits in their niches? An understanding of such behaviours might provide considerable insight into the biology of these fungi as well as providing potential targets for novel antifungal therapy.

## Declaration of Competing Interest

The authors have no competing interests.
